# Total Flavonoids of Rhizoma Drynariae Enhances Angiogenic-Osteogenic Coupling During Distraction Osteogenesis by Promoting Type H Vessel Formation Through PDGF-BB/PDGFR-β Instead of HIF-1α/ VEGF Axis

**DOI:** 10.3389/fphar.2020.503524

**Published:** 2020-11-27

**Authors:** Zhen Shen, Zehua Chen, Zige Li, Yan Zhang, Tao Jiang, Haixiong Lin, Minling Huang, Huamei Chen, Junjie Feng, Ziwei Jiang

**Affiliations:** ^1^ Department of Orthopaedics, Kunming Municipal Hospital of Traditional Chinese Medicine, The Third Affiliated Hospital of Yunnan University of Chinese Medicine, Kunming, China; ^2^ Department of Orthopaedics, the First Affiliated Hospital of Guangzhou University of Chinese Medicine, Guangzhou, China; ^3^ The Fifth Clinical Medical College, Guangzhou University of Chinese Medicine, Guangzhou, China

**Keywords:** PDGF-BB/PDGFR-β axis, angiogenic-osteogenic coupling, type H vessels, distraction osteogenesis, total flavonoids of drynariae rhizoma

## Abstract

**Background:** Total flavonoids of Rhizoma Drynariae (TFRD), extracted from the kidney-tonifying traditional Chinese medicine Rhizoma Rrynariae, has been proved to be effective in treating osteoporosis, bone fractures and defects. However, pharmacological effects of TFRD on type H vessels, angiogenic-osteogenic coupling in distraction osteogenesis (DO) and the mechanism remain unclear. This study aims at investigating whether type H vessels exist in the DO model, effects of TFRD on angiogenic-osteogenic coupling and further elucidating the underlying mechanism.

**Methods:** Rats models of DO and bone fracture (FR) were established, and then were separately divided into TFRD and control subgroups. Imageological and histological analyses were performed to assess bone and vessel formation. Immunofluorescent staining of CD31 and endomucin (Emcn) was conducted to determine type H vessel formation. Matrigel tube formation, ALP and Alizarin Red S staining assays were performed to test the effects of TFRD on angiogenesis or osteogenesis of endothelial precursor cells (EPCs) or bone marrow-derived mesenchymal stem cells (BMSCs). Additionally, expression levels of HIF-1α, VEGF, PDGF-BB, RUNX2 and OSX were determined by ELISA, qPCR or western blot, respectively.

**Results:** The *in vivo* results indicated more formed type H vessels in DO groups than in FR groups and TFRD obviously increased the abundance of type H vessels. Moreover, groups with higher abundance of type H vessels showed better angiogenesis and osteogenesis outcomes. Further *in vitro* experiments showed that TFRD significantly promoted while blocking PDGF-BB remarkably suppressed the angiogenic activity of EPCs under stress conditions. The levels of *p*-AKT and *p*-ERK1/2, downstream mediators of the PDGF-BB pathway, were up-regulated by TFRD but blocked by function blocking anti-PDGF-BB antibody. In contrast, the activated AKT and ERK1/2 and corresponding tube formation were not affected by the HIF-1α inhibitor. Besides, blocking PDGF-BB inhibited the osteogenic differentiation of the stretched BMSCs, but TFRD enhanced the osteogenic activity of BMSCs and ameliorated the inhibition, with more calcium nodes, higher ALP activity and mRNA and protein levels of RUNX2 and OSX.

**Conclusion:** Type H vessels exist in the DO model and TFRD enhances angiogenic-osteogenic coupling during DO by promoting type H vessel formation via PDGF-BB/PDGFR-β instead of HIF-1α/VEGF axis.

## 1. Introduction

Distraction osteogenesis (DO) becomes a unique and effective technique for bone regeneration in clinical practice, which has been widely applied in treating bone defects, limb shortening and reconstruction since reported by Ilizarov (Ilizarov and Soĭbel’man, 1969). The process of DO consists of three sequential phases, namely the latency, distraction and consolidation phase. Initially, bone segments are strictly fixed with the external fixation for 5–7 days after osteotomy. Then the bone segments proximal and distal to the osteotomy site are separated through gradual and continuous distraction. Finally, the external fixation is maintained in place without distraction until the newly-formed bone is so mechanically strong that the external fixation could be safely removed. (Ilizarov, 1988; Li et al., 2015). Although DO has become the first-line treatment method for bone defects and limb deformities, limitations such as prolonged treatment period and slow callus formation or mineralization have not been well solved yet, which inevitably places economic, physiological and psychological burdens on patients and even increases complications (Spiegl et al., 2013). Worse still, even though large numbers of studies tried to investigate the mechanisms of DO-induced neo-osteogenesis from the perspective of miRNAs (Sun et al., 2017a; Sun et al., 2017b), exosomes (Jia et al., 2019b) or various biological materials (Montes-Medina et al., 2018; Jia et al., 2019a), the detailed molecular mechanisms of DO in bone repair are still not well understood, which subsequently results in lack of pharmacotherapies that accelerate bone formation and improve bone quality. Therefore, accelerating callus formation and mineralization, shortening the consolidation duration and further investigating the underlying mechanisms during DO are of great clinical significance.

As is well known, bone is a highly vascularized tissue enriched in large vessels and capillaries (Tomlinson and Silva, 2013). Sufficient blood supply is beneficial to bone formation and angiogenesis is a prerequisite step in treating skeletal diseases such as bone fractures and defects (Stegen et al., 2015; Ramasamy et al., 2016). Growing studies have demonstrated that skeletal development and repair occur in close spatial and temporal association with angiogenesis (Brandi and Collin-Osdoby, 2006; Maes, 2013; Saran et al., 2014; Huang et al., 2016; Yang et al., 2017), which suggests the existence of signal transmission or crosstalk between osteoblastic or osteoprogenitor cells and vascular endothelial cells. The association between angiogenesis and osteogenesis is thought to be a close link called “angiogenic-osteogenic coupling” (Xie et al., 2014; Grosso et al., 2017). More importantly, recent studies reported by Kusumbe et al. (Kusumbe et al., 2014; Ramasamy et al., 2014) have discovered that the CD31^hi^Emcn^hi^ vessel, a specific vessel subtype called type H, strongly positive for CD31 and Emcn, is believed to possess specialized functional properties in local microenvironments. Type H vessels not only transport a source of growth factors and nutrients to the injured bone site, but also secrete active factors to directly regulate bone formation, which further enhance the communication between the vascular and osteogenic cells and promote the angiogenic-osteogenic coupling in bone (Yang et al., 2017; Bernard, 2018).

Angiogenesis has been confirmed during the DO process, especially in the distraction phase (Fang et al., 2005; Rachmiel and Leiser, 2014). Furthermore, stimulation of angiogenesis with various cytokines has yielded satisfactory osteogenesis during DO in animal models (Fujio et al., 2011; Davidson et al., 2011; Donneys et al., 2013), which indicates that angiogenic-osteogenic coupling also plays an important role in DO-induced bone regeneration. Considering the important role of type H vessels in angiogenic-osteogenic coupling, possibility of certain relation between type H vessels and DO may exist. Unfortunately, although type H vessels have been successively found in models of osteoporosis (Huang et al., 2018), fractures (Xu et al., 2018) and bone defects (Wang et al., 2017), it still remains unknown whether type H vessels exist in the DO model and whether regular distraction stress could stimulate type H vessel formation. Moreover, until now, there is little research on drugs that exert positive effects on type H vessel formation.

Total flavonoids of Rhizoma Drynariae (TFRD), the major component in the well-known kidney-tonifying traditional Chinese medicine Rhizoma Drynariae with pharmacological activities in animal experiments against osteoporosis (Sun et al., 2016), bone fractures (Zhang Y.L et al., 2017) and inflammation (Anuja et al., 2010), has been proved to exert multifaceted pharmacological effects on the skeleton system, such as up-regulating the expression levels of BMP-2 and Runx2 (Yao et al., 2018), promoting osteoblast proliferation and differentiation (Chen et al., 2011), inhibiting osteoclast activity and preventing bone loss (Guo et al., 2011). Nowadays, TFRD has been developed into a postmarketing Chinese medicine called Qianggu capsule (drug approval number: Z20030007, Qi-Huang Pharmaceutical CO. LTD., Beijing, China) (Sun et al., 2016). Our previous study has demonstrated that TFRD could accelerate bone formation and remodeling in the distraction gap (Shen et al., 2019). Moreover, TFRD was also found to have the potential to promote vascularization during DO (Shen et al., 2019). However, the underlying mechanism of the positive effects of TFRD on angiogenesis and osteogenesis during DO remains little clear.

In the light of dual properties of type H vessels in regulating angiogenesis and osteogenesis, from the perspective of angiogenic-osteogenic coupling, we proposed the hypothesis that type H vessels might also exist in the DO model and TFRD could promote type H vessel formation that would enhance the angiogenic-osteogenic coupling and subsequently facilitate callus formation and mineralization during DO. In this study, to better investigate effects of gradual and continuous tensile stress on type H vessel formation, we established the fracture model as a control. First, we observed whether type H vessels existed in the rat DO model and investigated effects of TFRD on the bone and vessel formation in the rat DO and FR models. Then, *in vitro* experiments were conducted to examine effects of TFRD on EPCs and BMSCs under stress or non-stress conditions that simulate distraction osteogenesis or bone fracture states. Further, we investigated the potential mechanism of TFRD-promoted type H vessel formation during DO.

## 2. Materials and Methods

### 2.1. Drugs and Reagents

Total flavonoids of Rhizoma drynariae (TFRD) were purchased from Beijing Qihuang Pharmaceutical Manufacturing Co., Ltd. (National Medicine Permit No. Z20030007, number of production: 04080081, the content of TFRD ≥ 80%). Reagents associated with hematoxylin and eosin (H&E), masson’s trichrome (Masson’s), safranin O-fast green (Safranin O) and immunohistochemical (IHC) analyses were obtained from Google Biotechnology Limited Company (Wuhan, China). All the supplementary components of cell culture were from Invitrogen Gibco (USA). Anti-CD31 and anti-endomucin antibodies were purchased from Santa Cruz (USA). Anti-RUNX2, anti-OSX, function blocking anti-PDGF-BB antibodies and the Hif-1α inhibitor were from Abcam (USA). Specific antibodies against AKT, phospho-AKT (Ser473), ERK, phospho-ERK (Thr202/Tyr204) were purchased from CST (USA). Hif-1α, PDGF-BB and VEGF ELISA kits were purchased from Sinoukbio (Beijing, China). EGM-2 MV medium and mesenchymal stem cell growth medium were purchased from Lonza (USA). Cell Counting Kit-8 (CCK-8) kit, Alkaline Phosphatase Assay (ALP) kit, RIPA and TBS/Tween-20 solution were purchased from Beyotime Biotechnology (Shanghai, China). ALP activity detection kit was purchased from Jiancheng Bioengineering (Nanjing, China) and Alizarin Red S was from Sigma (USA). Matrigel was purchased from Corning (USA). PrimeScript RT reagent and SYBR Green qRT-PCR kits were from TakaRa (Japan).

### 2.2. Animals and Ethical Approval

A total of forty-eight 12-week-old male Sprague-Dawley rats (weighting 280–320 g) were kept in the laboratory with standard conditions at 24°C under 12:12 h light-dark cycle and fed with a standard diet. All animal care and experimental procedures were approved by the Institutional Animal Ethics Committee of the First Affiliated Hospital of Guangzhou University of Traditional Chinese Medicine (ethical approval number: TCMF1- 2018002).

### 2.3. Experimental Design

All rats were randomly and averagely assigned to one of the two groups: distraction osteogenesis (DO) group (rats subjected to DO operation) and bone fracture (FR) group (rats subjected to fracture operation). And either of the two groups were then divided into two subgroups including TFRD subgroup (according to the previous study (Song et al., 2016), rats administered orally with TFRD at a dose of 75 mg/kg body weight/day) and control subgroup (rats orally fed with an equal amount of vehicle) (n = 12 per subgroup). From the first day after surgery, rats in the TFRD group were orally fed with TFRD until the end of the experiment.

**FIGURE 1:**
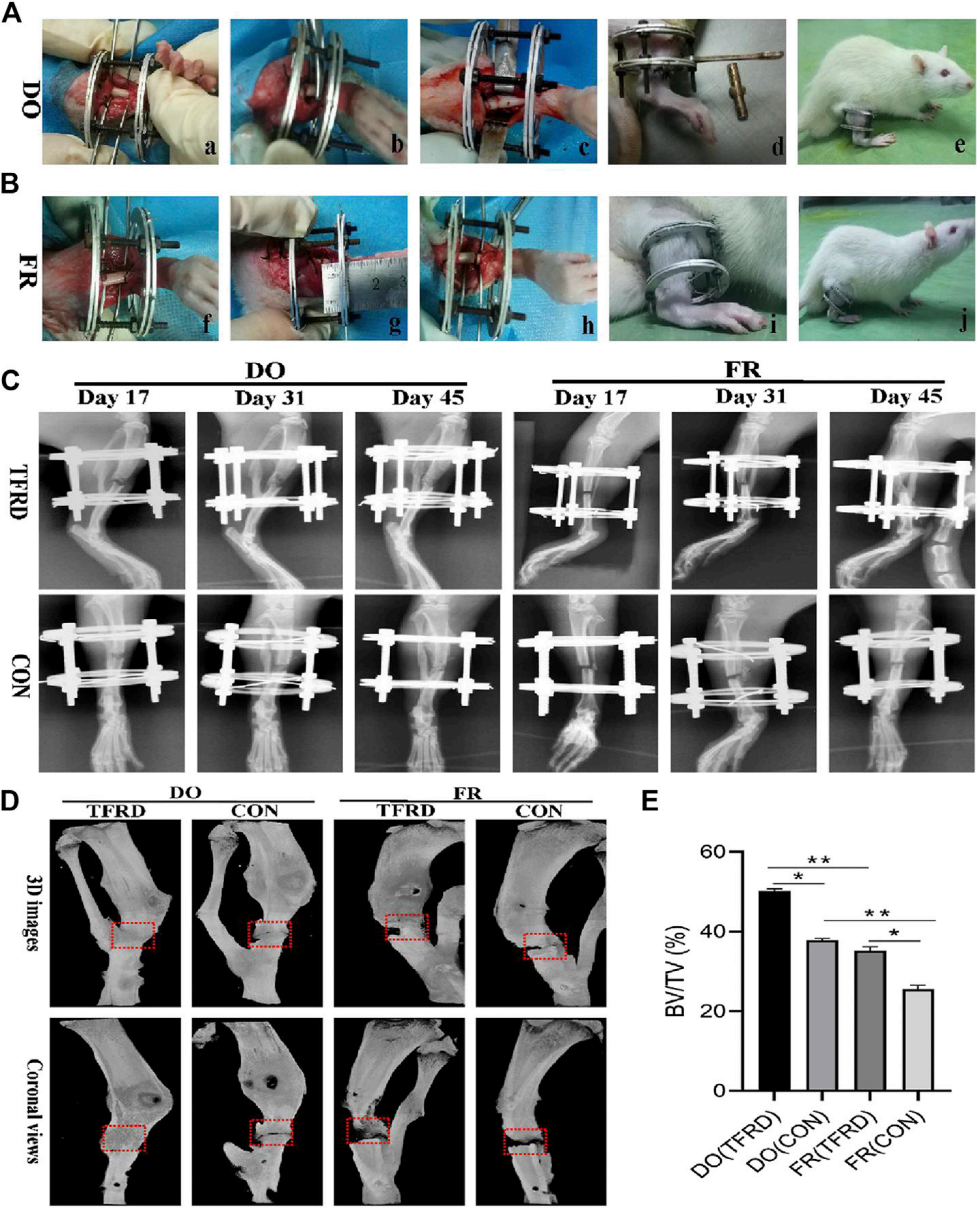
Surgical procedures and imageological manifestations of DO and FR models. **(A)**, **(B)** Creation of DO and FR models. a) tibia exposure and external fixator fixation; b) osteotomy; c) shortening and aligning the osteotomy ends; d) distraction procedure; e) consolidation stage; f) tibia exposure and external fixator fixation; g) osteotomy; h) not shortening the osteotomy ends; i) no distraction procedure; j) consolidation stage. **(C)** X-ray photographs obtained at 17, 31 and 45 days after surgery (n = 9 per group, n = 3 per time point). **(D)** Representative micro-CT images of bone regeneration in DO (TFRD) group, DO (CON) group, FR (TFRD) group and FR (CON) group at 45 days after surgery. Red dotted boxes indicate region of interest (ROI), representing bone defect areas. **(E)** Quantification of bone tissue volume/total tissue volume (BV/TV) in the four groups. Significant difference is present when *p < 0.05,* displayed with an asterisk, or *p < 0.01,* displayed with two asterisks, while NS represents no significant difference.

DO and FR models were established as described previously (Shen et al., 2019). In brief, after the rats were generally anesthetized with intraperitoneal pentobarbital (3 mg/100 g, Sigma, St. Louis, MO, USA), a longitudinal incision was made in the skin distal to the right tibia crest and the bone was exposed. Meanwhile, surgical scissors were used to snip the fibula. Then a custom-made circle external device was assembled and fixed to the tibia by four 27-gauge stainless steel needles (Baokang, Zhangjiagang, China). After stabilization, transverse corticotomies using a Gigli saw (Baokang, Zhangjiagang, China) were performed to create a 2 mm long diaphyseal defect on the tibia. For DO group, the two osteotomy surfaces were shortened and brought into close apposition, which was followed by a 7 days latency period. Subsequently, the distraction procedure was initiated at a rate of 0.1 mm per 12 h until the length of osteotomy was restored, while the 2-mm gap was maintained by the external device throughout this study in the FR group (Figures 1A,B). Finally, buprenorphine (1.0 mg/kg), as an analgetic, was given by intramuscular injection for 3 days.

### 2.4. X-Ray, Micro-CT and Angiography Analyses

The consecutive X-ray photographs were obtained on days 17, 31 and 45 after surgery to show the dynamic healing process of bone (n = 3 per time point). After X-ray examination, the three rats of per group at day 17 after surgery were euthanized and perfused with Microfil (Microfil MV-122, Flow Tech; Carver, MA, USA). Briefly, the rib cage was opened after anesthetization, the descending aorta was clamped, and the inferior vena cava was incised. Subsequently, the vasculature was flushed with 0.9% normal saline containing heparin sodium (100 U/mL) and 20 ml of Microfil were respectively perfused into the left ventricle with an angiocatheter. Subsequently, the rats were stored at 4°C overnight to ensure polymerization of the contrast agent, after which the tibias were dissected, fixed in 4% paraformaldehyde for 48 h, decalcified in 10% EDTA for four weeks and then imaged by micro-CT. In addition, the three rats of per group at day 45 after surgery were scanned with micro-CT (SkyScan 1076, Kontich, Belgium) at a resolution of 20 μm (70kV and 130 µA radiation source with 0.5 mm aluminum filter). The bone tissue volume/total tissue volume (BV/TV) inside the defect gaps were determined using Scanco software. All tests were repeated with three specimens.

### 2.5. Histological, Immunohistochemical and Immunofluorescent Analyses

Tibia specimens were taken 17 and 45 days after surgery, decalcified in 10% EDTA for 4 weeks, dehydrated through graded ethanol of increasing concentration and then embedded in paraffin. Subsequently, the specimens were cut into 5 μm thick longitudinally oriented sections, of which some were processed for H&E, Masson’s and Safranin O staining (5 random visual fields per section, three sections per staining, nine sections per rat and three rats per time point).

Other sections were for immunohistochemical and immunofluorescent staining. Briefly, some sections were antigen retrieved, incubated with anti-CD31 (sc-71873, Santa Cruz, USA) primary antibody and then incubated with corresponding biotinylated secondary antibody. Blood vessels were defined with the positive CD31 staining and their typical round or oval structure. Meanwhile, the rest sections were subjected to double immunofluorescent staining for CD31 (sc-71873, Santa Cruz, USA) and Emcn (sc-19901, Santa Cruz, USA), which were conducted to determine and evaluate type H vessel formation, as previously described (Wang et al., 2017). Briefly, bone sections were stained with individual primary antibodies to mouse CD31 and EMCN overnight at 4°C and then stained with secondary antibodies conjugated with fluorescence at room temperature for 1 h. Nuclei were stained with DAPI. Images were acquired with a Leica DMI6000B fluorescence microscope (Solms, Germany). The results were quantified using Image J software. All tests were repeated with three specimens.

### 2.6. Cell Culture and Identification of EPCs and BMSCs

EPCs and BMSCs were cultured following previous techniques (Zhang M. et al., 2017), with minor modifications. Briefly, the bone marrow cavities of femurs and tibias were washed at 4°C in 0.01 M PBS. Mononuclear cells were collected from marrow suspension and then cultured with EGM-2 MV medium (Lonza, USA) for EPC culture, or cultured in mesenchymal stem cell growth medium (Lonza, USA) for BMSC culture. After 24 h for culture, the attached cells were plated into 50 ml glass flask coated with fibronectin (Sigma, USA) at a density of 1.0 × 10^6^/L and the medium was changed every 3 days. Cells at passage three were used for the following experiments. For EPC and BMSC identification, expressions of specific markers for EPCs and BMSCs were assessed via immunofluorescent staining as described previously (Zhang et al., 2017). Briefly, EPCs were identified by ulex europaeus agglutinin-1 lectin (UEA-1 lectin)^+^, CD31^+^ and CD34^+^ markers using immunofluorescent staining, while BMSCs were identified by α-smooth muscle actin (α-SMA)^+^ and CD29^+^ markers. Cells from passage three were used in this study (Supplementary Figure S1). In addition, for the surface marker and purity identification and differentiation capacities of BMSCs, a BMSC suspension (1 × 10^6^ cells/mL) was prepared and washed twice with PBS, then incubated in the dark at room temperature with primary antibodies against CD44 (FITC-labeled), CD90 (PE-labeled), CD31 (FITC-labeled), CD45 (FITC-labeled) and isotype-matched normal IgG were used as controls (all antibodies were purchased from BD Biosciences, USA). After incubation, cells were washed with PBS and immediately subjected to flow cytometry analysis with a flow cytometer (BECKMAN, USA) to determine the cell surface marker expression and purity of BMSCs. For osteogenic, adipogenic and chondrogenic differentiation, cells were detected with Alizarin red S, Oil Red O and toluidine blue staining kits under osteogenic, adipogenic and chondrogenic induction conditions for 14 days, respectively (Supplementary Figure S2).

### 2.7. Establishment of Stress Conditions

As described previously (Naruse et al., 1998), the stress conditions were established using the STREX cell stretching system (ST-160, B-Bridge, USA) to apply regular tensile stress to cells in a single, parallel direction at an approximate ratio of 10%.

### 2.8. Preparation of the Conditioned Medium

EPCs were seeded (4 × 105 cells per well) in 6-well culture plates and cultured in EGM-2 MV medium with 100 μg/ml TFRD dissolved in dimethylsulfoxide (DMSO) or an equal volume of vehicle (DMSO) for 3 days under stress conditions. After 3 days of induction, the conditioned medium (CM) from the stretched EPCs exposed to 100 μg/ml TFRD or DMSO as a control were collected by centrifugation (2000 r.p.m., 10 min) for osteogenesis-related assays of BMSCs.

### 2.9. Cell Viability Assay

EPCs were exposed to different doses of TFRD dissolved in DMSO (0, 12.5, 25, 50, 100 and 200 ug/mL) or DSMO as the vehicle for 24, 48 and 72 h, respectively. The concentration of DMSO used in the present study was ≤0.1% (v/v), namely ≤1 μL/ml. EPCs (5 × 10^3^ cells per well) were seeded onto 96-well plates and cultured in EGM-2 MV medium, in which the final concentration of FBS is 5% (v/v). Each well was subjected to 10 μL CCK-8 solution for a further 10 min at 37°C. The absorbance value of each well was measured at 450 nm using a microplate reader (Bio-Rad 680). The study was performed in triplicate.

### 2.10. Enzyme-Linked Immunosorbent Assay (ELISA)

The concentrations of HIF-1α, PDGF-BB and VEGF in serum and PDGF-BB in the cell supernatant were measured using commercial rat ELISA kits (Sinoukbio, Beijing, China) according to the manufacturer’s instructions. The optical density of each well was determined using a microplate reader (Bio-Rad 680, Hercules, USA) at 450 nm.

### 2.11. Matrigel Tube Formation Assay

Matrigel tube formation assay was performed according to the previous protocol (Matluobi et al., 2018), with minor modifications. Briefly, Matrigel growth factor reduced (GFR) basement membrane matrix (356230, Corning, USA) was used to assess tube formation of EPCs, which was thawed on ice and 100 μL matrigel was applied to each well at 37°C for 30 min according to the manufacturer’s instructions. Then EPCs (6 × 10^4^ cells per well) were seeded onto Matrigel-coated 24-well plates and cultured with or without TFRD (100 μg/ml) at 37°C and 5% CO_2_ for 6 h after a 1-h stimulation of function blocking anti-PDGF-BB antibody (20 μg/ml) or not. To confirm the role of the PDGF-BB/PDGFR-β and HIF-1α/VEGF axis in angiogenesis, EPCs were pretreated with function blocking anti-PDGF-BB antibody (20 μg/ml) or HIF-1α inhibitor YC-1 (10 μM) for 1 h prior to TFRD addition. After incubation for 6 h, cells were observed with an inverted microscope (Leica, Wetzlar, Germany). Total tube length, total branching points and total loops in five randomly chosen fields were quantified by Image J software. The study was performed in triplicate.

### 2.12. Osteogenic Differentiation and ALP/Alizarin Red S (ARS) Staining

The medium was removed and replaced by osteogenic induction medium (1 nM dexamethasone, 50 mM L-ascorbic acid-2-phosphate and 20 mM β-glycerolphosphate with complete medium), supplemented with CM (TFRD) or CM (DMSO). The induction medium was changed every 3 days. After 7 and 14 days for induction, ALP staining at 7 days of induction and Alizarin Red S staining at 14 days of induction were performed separately to evaluate positive rate of alkaline phosphatase and calcium deposit formation, in which ALP staining and ALP activity were measured by the Alkaline Phosphatase Assay Kit (Beyotime, Shanghai, China) and ALP activity detection kit (Jiancheng Bioengineering, Nanjing, China). All the experiments were repeated three times independently.

### 2.13. Quantitative Real-Time Polymerase Chain Reaction (qPCR)

BMSCs were seeded (2 × 10^5^ cells/well) in 12-well plates and preincubated with or without function blocking anti-PDGF-BB antibody (20 μg/ml) overnight. Then BMSCs were stimulated with the CM (TFRD) or CM (DMSO) from the stretched EPCs for 7 days. The qPCR analysis was performed using the Prime Script^TM^RT reagent Kit SYBR (Takara, DRR047A) followed by Premix EX Taq Ⅱ Kit (TaKaRa, Japan, RR820A). The reaction run at one cycle of 95°C for 3 min, followed by 40 cycles of 95°C for 5 s, 60°C for 30 s β-Actin was used as the internal control. Primer sequences of RUNX2, Osteorix and β-actin, were summarized in Supplementary Table S1. The data were analyzed using the CT 2^−ΔΔCt^ method and expressed as a fold change respective to the control. The results were repeated three times independently.

### 2.14. Western Blot Analysis

EPCs were seeded (6 × 10^4^ cells/well) onto the 24-well plates. EPCs were pretreated with function blocking anti-PDGF-BB antibody (20 μg/ml) or HIF-1α inhibitor YC-1 (10 μM) for 1 h, and then treated with or without TFRD (100 μg/ml) for 6 h. BMSCs were seeded (2 × 10^5^ cells/well) in 12-well plates. Subsequently, the cells were preincubated with or without function blocking anti-PDGF-BB antibody (20 μg/ml) overnight, and then cultured with the CM(TFRD) or CM(DMSO) for 7 days. All the proteins were obtained from cultured cells by using RIPA (Beyotime, Shanghai, China) supplemented with 100 mM phenylmethanesulfonyl fluoride (Beyotime, Shanghai, China), protease inhibitors (Beyotime) and phosphatase inhibitors (Beyotime). After the 15 min centrifugation at 12,000 rpm, supernatants were extracted. Proteins were resolved on 10% SDS-PAGE and transferred by electroblotting to PVDF membranes (Millipore, USA). Membranes were then blocked in 5% (w/v) nonfat dry milk in TBS/Tween-20 solution (Beyotime), at room temperature for 45 min, followed by incubation with indicated antibodies (1:1,000 dilution) at 4°C overnight. After washing 5 times with TBS/Tween-20, bands were then incubated with the horse-radish peroxidase (HRP) conjugated anti-mouse or anti-rabbit secondary antibodies (1:5,000 dilution) for 1 h at room temperature. The protein levels were normalized against β-actin. Representative bands were selected from the three independent experiments.

### 2.15. Statistical Analysis

All data were presented as the mean ± standard error (SEM). Differences among groups were assessed by one-way analysis of variance and paired t-tests were conducted for comparisons between two methods in one group. SPSS 19.0 software (SPSS, Chicago) was used to analyze experimental data. The differences among four groups were considered to be statistically significant if *p* < 0.05.

## 3. Results

### 3.1. Intragastric Administration of TFRD Facilitates Bone Healing by Promoting Bone and Vessel Formation in DO and FR Rats

**FIGURE 2:**
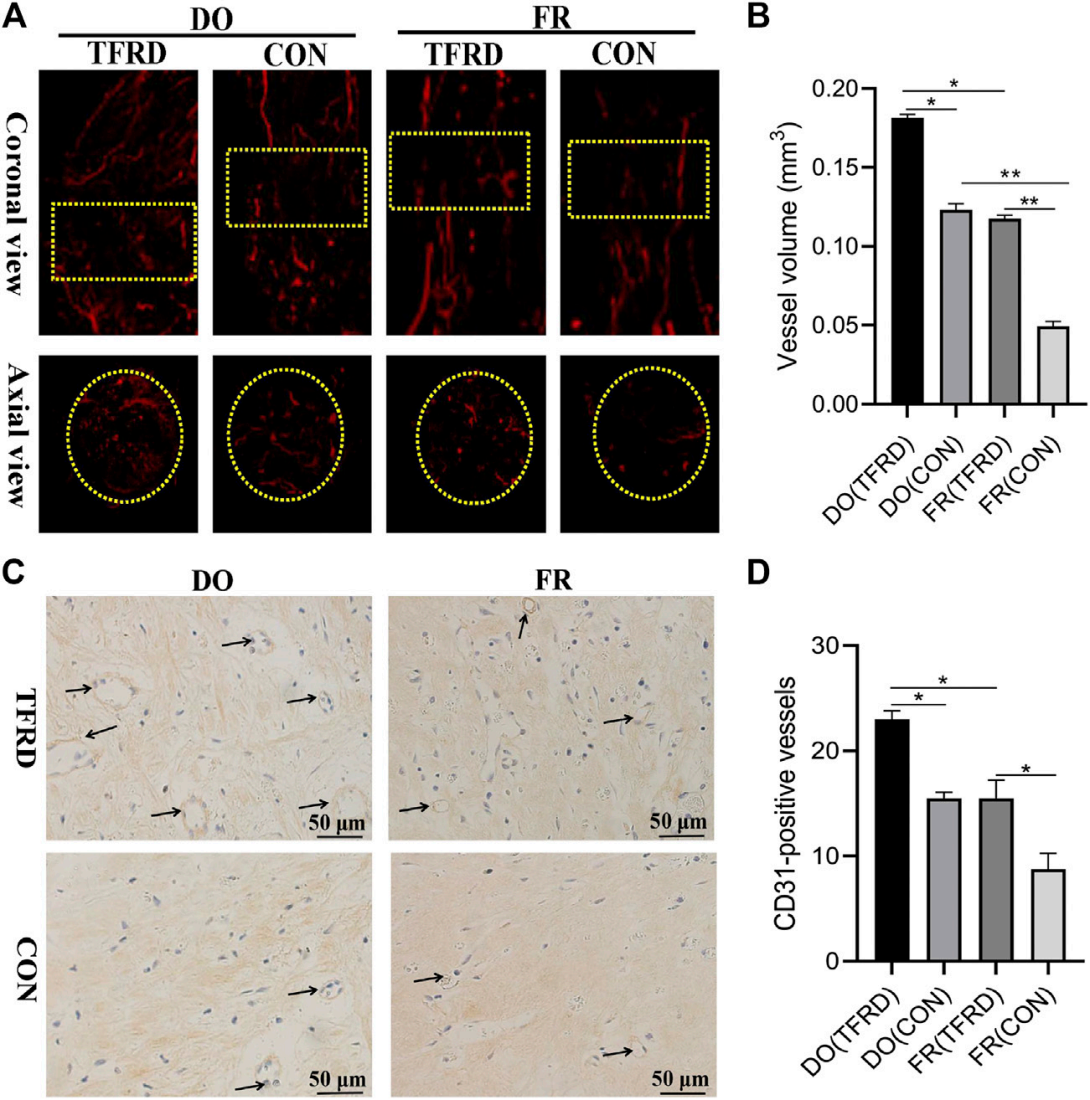
Effects of TFRD on vessel formation in rats of DO and FR models. **(A)** Representative angiographic images of four groups at 17 days after surgery in both coronal (top panel) and axial (lower panel) views. Yellow dotted boxes indicate region of interest (ROI) (n = 3 per group). **(B)** Quantification of the vessel volume at **(A)**. **(C)** CD31 expression inside the bone fracture or distraction regions of four groups obtained by immunohistochemistry staining at 17 days after surgery (n = 3 per group). **(D)** Quantification of CD31-positive vessels in bone fracture or distraction zone. Black arrows indicate CD31-positive vessels. The data are expressed as the mean ± SEM of three independent experiments, *p < 0.05, **p < 0.01, NS: not significant.

**FIGURE 3:**
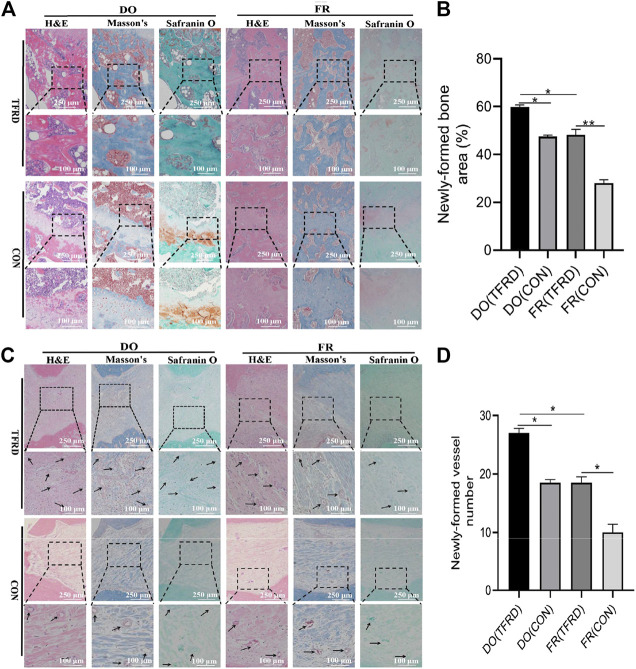
TFRD accelerates bone healing by promoting bone and vessel formation in rats of DO and FR models. **(A)** Representative histological images and **(B)** quantification of newly-formed bone at 45 days after surgery (n = 3 per group). **(C)** Representative histological images and **(D)** quantification of newly-formed vessels at 17 days after surgery (5 random visual fields per section, three sections per staining, nine sections per rat and three rats per time point). From left to right: H&E, Masson’s and Safranin O staining. The black boxes represent higher-magnification view. The data are expressed as the mean ± SEM of three independent experiments; Black arrows indicate micro-vessels. *p < 0.05, **p < 0.01, NS: not significant.

The X-ray and Micro-CT results indicated more newly-formed callus and earlier bone union in DO groups than in FR groups over time. In addition, TFRD administration obviously facilitated the callus formation and union compared to control (Figures 1C–E). Similar to the style of the new bone formation, the angiography analysis demonstrated that more vessels were observed in DO groups than in FR groups and TFRD resulted in a remarkable increase in vessel formation compared with control (Figures 2A,B). Moreover, the immunohistochemistry result also verified that TFRD could apparently up-regulated the expression of CD31 and there were more CD31-positive vessels in DO groups than in FR groups (Figures 2C,D). Additionally, the findings above were further confirmed by histological analyses. As shown by HE, Masson’s and Safranin O staining, most fibrous connective tissues were observed inside the bone fracture regions with little bone formation in FR (CON) group at 45 days after surgery, while moderate immature new bone formation appeared inside the distraction regions in DO (CON) group (Figure 3A). More importantly, bone formation in both DO and FR groups was found to be significantly promoted by TFRD. Especially, the DO (TFRD) group had higher percentage of newly-formed bone area and achieved better bone connection and integration with mineralized bone tissue bridging distraction gap than FR (CON) group (Figure 3B). Likewise, new vessel formation in the HE, Masson’s and Safranin O stained sections of all groups almost showed the similar pattern as bone formation (Figures 3C,D). Hence, it was identified that TFRD exerted positive effects on both osteogenesis and angiogenesis.

### 3.2. TFRD Increases the Abundance of Type H Vessels During the Process of Bone Healing

**FIGURE 4:**
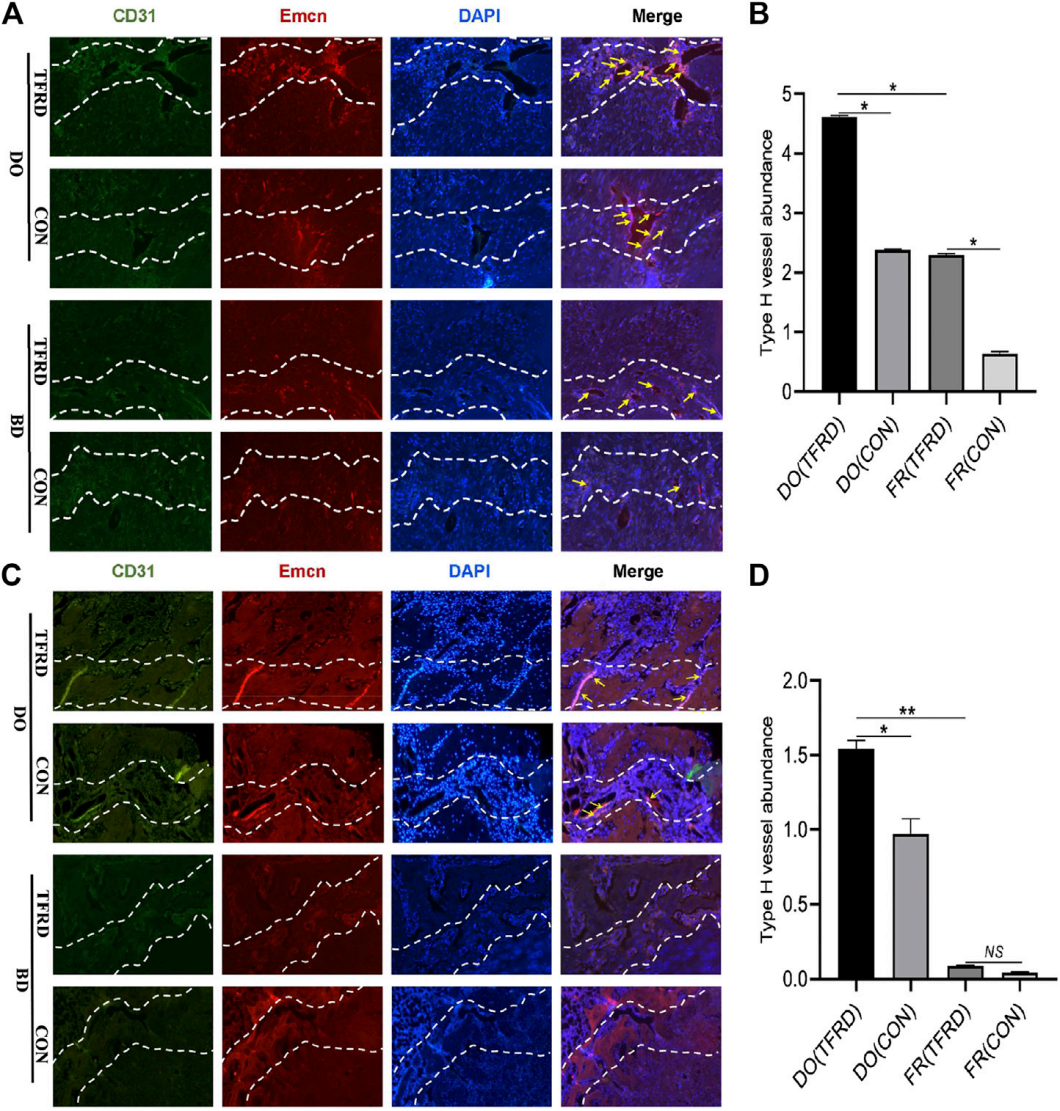
TFRD increases the abundance of type H vessels during bone healing process. **(A)** Representative immunostaining images of CD31 (green), Emcn (red) and Nuclei (blue, stained with DAPI) inside bone fracture or distraction regions of four groups at 17 and **(C)** 45 days after surgery (n = 3 per time point). The region between the two white dotted lines represents the bony gaps including bone fracture or distraction zone. The distraction zone was caused by gradual and controlled tensile stress. The type H vessels were marked with yellow arrows. **(B)**, **(D)** Quantification of the abundance of CD31^+^Emcn^+^ (type H) vessels (light pink) at 17 and 45 days after surgery, respectively. The abundance of type H vessels is represented by the percentage of CD31^+^Emcn^+^ vessel area inside the bony gap. The data are expressed as the mean ± SEM of three independent experiments, *p < 0.05, NS: not significant.

**FIGURE 5:**
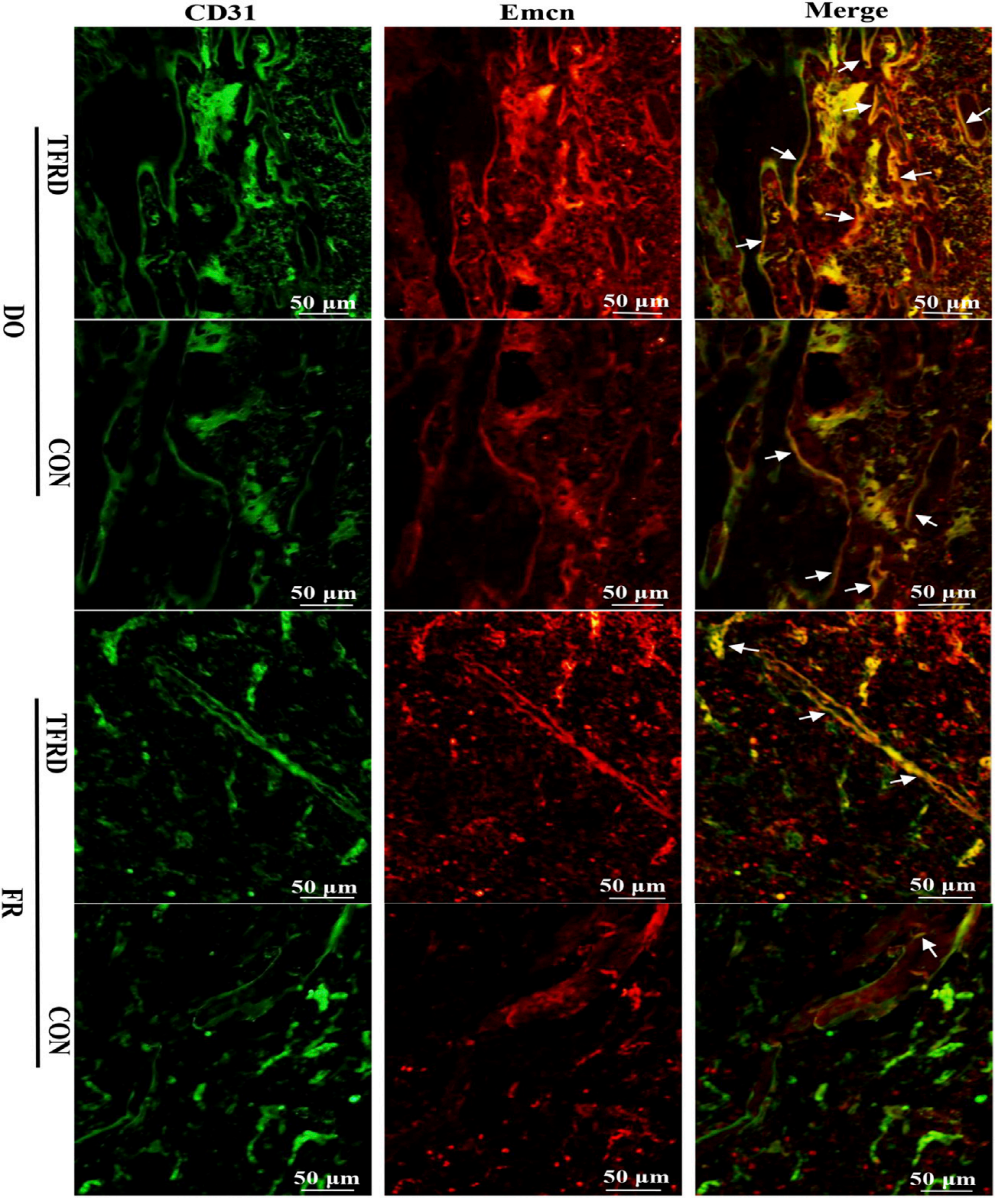
TFRD promotes the formation of type H vessels in both DO and FR models. White arrows indicate type H vessels.

As shown in Figure 4A and 4C, CD31 and Emcn immunofluorescence staining results demonstrated the presence of type H vessels in DO models and a higher proportion of type H vessels were observed in DO groups than in FR groups at 17 (Figure 4B) and 45 (Figure 4D) days after surgery, which indicated that regular tensile stress could stimulate the formation of type H vessels. Furthermore, the abundance of type H vessels was markedly increased by the addition of TFRD. Of note, the formation of type H vessels in DO and FR groups decreased over time and especially at 45 days after surgery almost no type H vessels were detected in FR (CON) group. However, administration of TFRD to rats led to an increase in the abundance of type H vessels and ameliorated the decline in abundance, which was clearly demonstrated in the images with a higher magnification (Figure 5). Together, these findings suggested that administration of TFRD could enhance the abundance of type H vessels during the process of bone healing.

### 3.3. TFRD Increases the Number of EPCs and the Production of PDGF-BB in a Dose-dependent Manner

**FIGURE 6:**
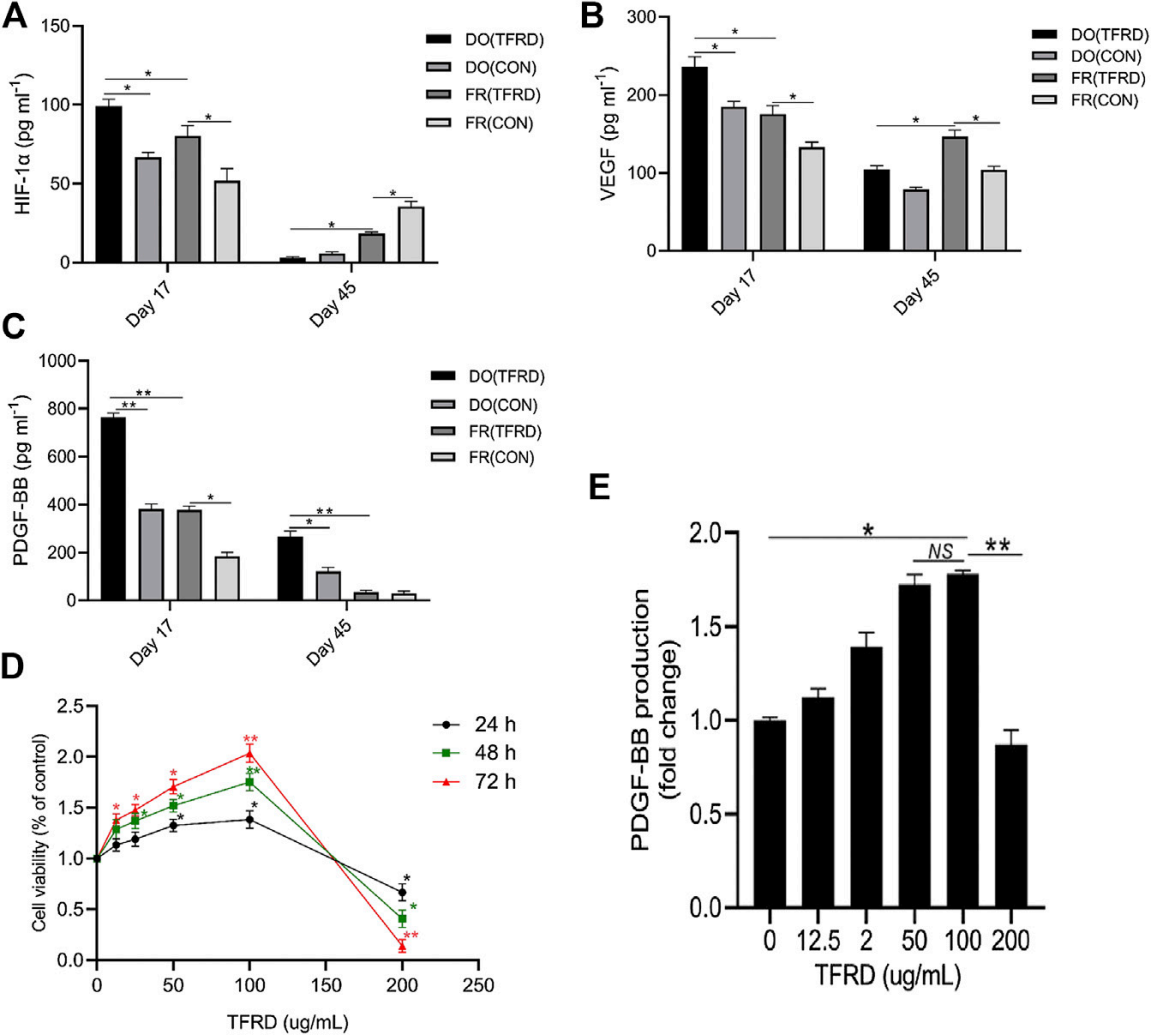
TFRD increases the number of EPCs and the production of PDGF-BB in a dose-dependent manner. **(A)**–**(C)** HIF-1α, VEGF and PDGF-BB concentrations in serum of DO and FR groups detected by ELISA (n = 3 per group). **(D)** The CCK-8 analysis of EPCs exposed to various concentrations of TFRD (0, 12.5, 25, 50, 100 and 200 μg/ml) for 24, 48 and 72 h, respectively. **(E)** Dose-dependent changes in PDGF-BB production from EPCs in response to TFRD treatment of different concentrations. The data are expressed as the mean ± SEM of three independent experiments, *p < 0.05, **p < 0.01, NS: not significant.

As demonstrated in Figures 6A and 6B, expressions of serum HIF-1α and VEGF in DO (CON) group were higher than those in FR (CON) group at 17 days after operation. Moreover, the levels of HIF-1α and VEGF were markedly up-regulated by TFRD, which was consistent with immunofluorescence results showing that more newly-formed type H vessels were detected in DO groups than in FR groups and TFRD remarkably promoted type H vessel formation (Figures 4A,B). But at 45 days after operation, HIF-1α and VEGF contents in DO groups were much lower than those in FR groups, while abundance of type H vessels in DO groups was higher than that in FR groups at the same time point (Figures 4C,D). In contrast, PDGF-BB expression was higher in DO groups than in FR groups at both 17 and 45 days after surgery (Figure 6C), which was significantly increased by administration of TFRD. These findings indicated that only PDGF-BB in expression level showed a similar trend when referring to the temporal change characteristics of type H vessels in DO and FR groups. Furthermore, the *in vitro* results also confirmed that the viability of EPCs and PDGF-BB secretion from EPCs were significantly enhanced in a dose-dependent manner after TFRD treatment, and the viability of EPCs and the level of PDGF-BB were the highest when the dose of TFRD was 100 μg/ml. Thus, the optimal TFRD concentration was 100 μg/ml (Figures 6D,E).

### 3.4. PDGF-BB/PDGFR-β Rather Than HIF-1α/VEGF Axis Involves in the TFRD-Promoted Angiogenesis Under Stress Conditions

**FIGURE 7:**
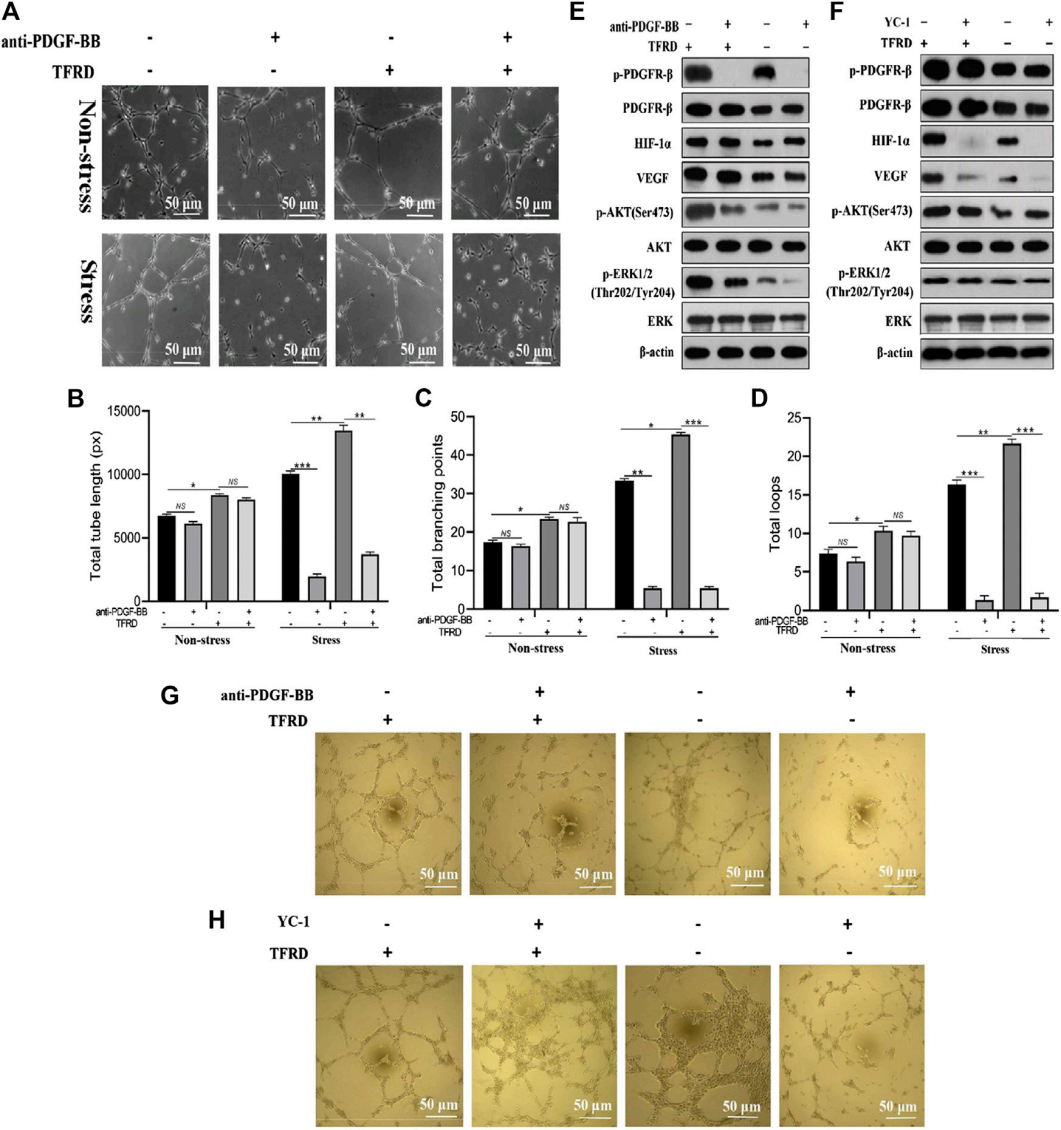
PDGF-BB/PDGFR-β instead of HIF-1α/VEGF axis involves in the TFRD-promoted angiogenesis under stress conditions. **(A)** Representative images of tube formation of EPCs stimulated with 100 μg/ml TFRD or 20 μg/ml function blocking anti-PDGF-BB antibody for 6 h under stress or non-stress conditions. **(B)** Quantification of total tube length, **(C)** total branching points and **(D)** total loops of EPCs in five randomly chosen fields. **(E)** Protein levels of *p*-PDGFR-β, HIF-1α, VEGF, p-PI3K and *p*-ERK1/2 in the stretched EPCs treated with or without TFRD for 6 h after the stimulation with 20 μg/ml function blocking anti-PDGF-BB antibody or **(F)** 10 μM HIF-1α inhibitor (YC-1) for 1 h. **(G)** Representative images of tube formation of the stretched EPCs stimulated with or without TFRD for 6 h after the stimulation with 20 μg/ml function blocking anti-PDGF-BB antibody or **(H)** 10 μM HIF-1α inhibitor (YC-1) for 1 h. The data are expressed as the mean ± SEM and all experiments were performed at least three times. *p < 0.05, **p < 0.01, ***p < 0.001, NS: not significant.

As presented in Figures 7A–D, TFRD-treated EPCs formed many tubes with obvious structures and total tube length, total branching points and total loops of EPCs were markedly increased under both stress and non-stress conditions. But interestingly, under non-stress conditions, blocking PDGF-BB almost caused no significant difference in angiogenesis of EPCs, while under stress conditions, the angiogenic activity of EPCs was remarkably suppressed by function blocking anti-PDGF-BB antibody. To further investigate whether PDGF-BB rather than HIF-1α mediated the TFRD-promoted angiogenesis under stress conditions, the stretched EPCs were precultured with 20 μg/ml function blocking anti-PDGF-BB antibody or 10 μM YC-1 (the HIF-1α inhibitor) for 1 h, followed by the treatment with or without 100 μg/ml TFRD. As shown in Figures 7E and 7G, TFRD markedly up-regulated the expressions of *p*-PDGFR-β, HIF-1α, VEGF, *p*-AKT and *p*-ERK1/2, however, the effect was blocked by function blocking anti-PDGF-BB antibody and the corresponding tube formation was also suppressed, while the activated AKT and ERK1/2 and the corresponding tube formation were not affected by the addition of HIF-1α inhibitor (Figures 6F,H).

### 3.5. TFRD Enhances PDGF-BB From EPCs Mediated Osteogenic Differentiation of BMSCs Under Stress Conditions

**FIGURE 8:**
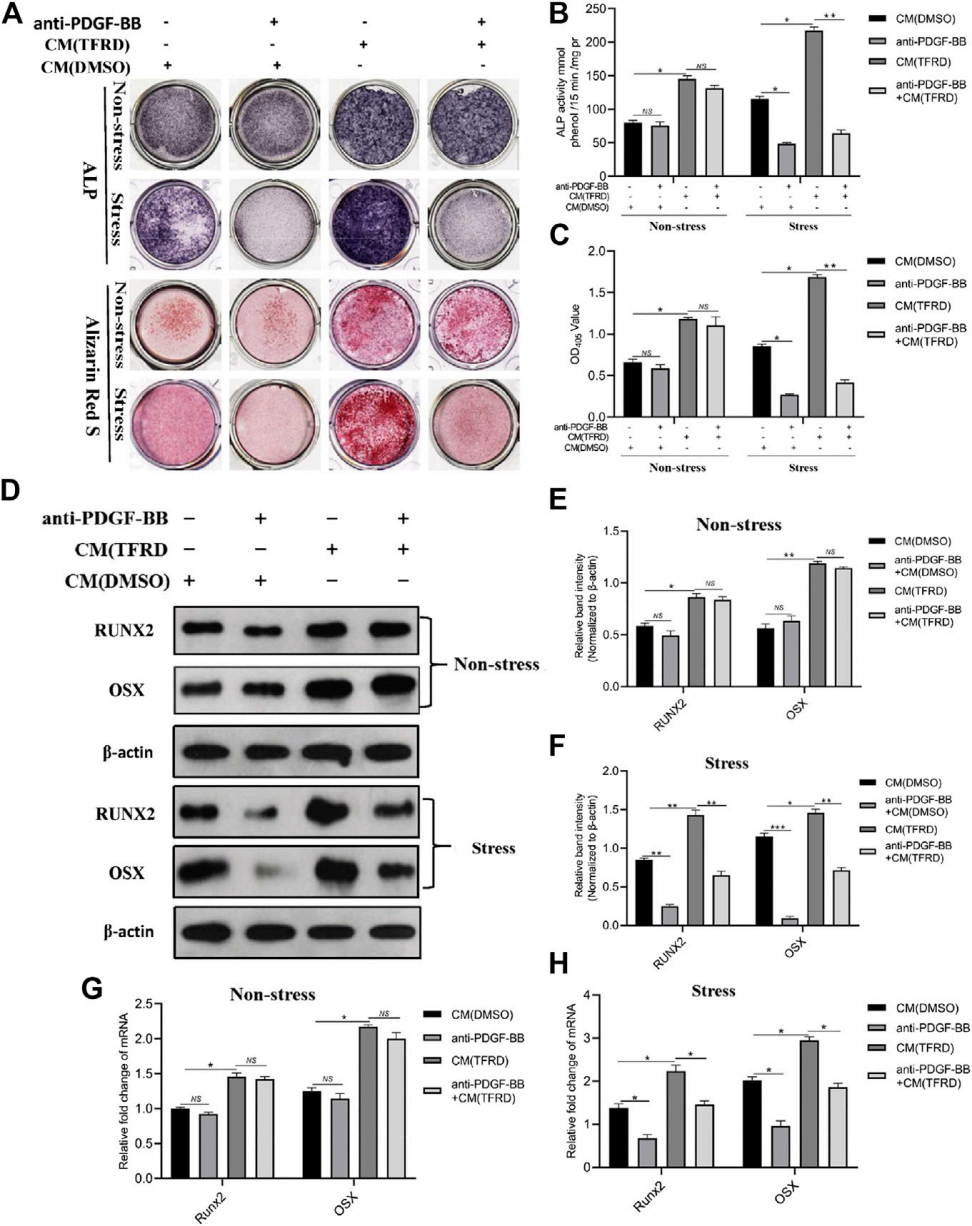
TFRD promotes the PDGF-BB-mediated osteogenic differentiation of BMSCs under stress conditions. **(A)** Representative images of ALP and ARS staining of the stretched or unstretched BMSCs at 7 and 14 days, respectively. **(B)**, **(C)** Quantitative determination of ALP activity and the production of mineralized nodules. **(D)** Representative images and **(E)**, **(F)** quantification of the protein levels of RUNX2 and OSX in the stretched or unstretched BMSCs treated with CM (TFRD) or CM (DMSO) for 7 days, following the exposure to 20 μg/ml function blocking anti-PDGF-BB antibody overnight. **(G)**, **(H)** The mRNA expression levels of RUNX2 and OSX in the stretched or unstretched BMSCs treated with CM (TFRD) or CM (DMSO) in the presence or absence of function blocking anti-PDGF-BB antibody 7 days after osteogenic differentiation. The data are expressed as the mean ± SEM. All experiments were performed at least three times. *p < 0.05, **p < 0.01, ***p < 0.001, NS: not significant.

Conditioned media (CM) from EPCs pretreated with 100 μg/ml TFRD or an equal volume of DMSO were applied to BMSCs to investigate the presence of cell to cell communication between angiogenic and osteogenic cells during the process of bone formation. As demonstrated in Figures 8A–C, compared to the CM (DMSO), the CM (TFRD) treatment resulted in higher ALP activity and more calcium deposition under both stress and non-stress conditions. But only under stress conditions, blocking PDGF-BB remarkably suppressed the TFRD-enhanced osteogenic capacity of BMSCs, while it exerted little influence on the osteogenic activity of BMSCs and no difference was observed under non-stress conditions. Consistent with ALP and ARS staining results, the qPCR and western blot results demonstrated that levels of RUNX2 and OSX were significantly up-regulated in the CM (TFRD) group compared with the CM (DMSO) group under both stress and non-stress conditions (Figures 8D–H). Similarly, blocking PDGF-BB obviously attenuated the TFRD-induced increase in the expressions of RUNX2 and OSX at the mRNA and protein levels under stress conditions alone (Figures 8F,H), but no significant difference was found under non-stress conditions (Figures 8E,G).

**FIGURE 9:**
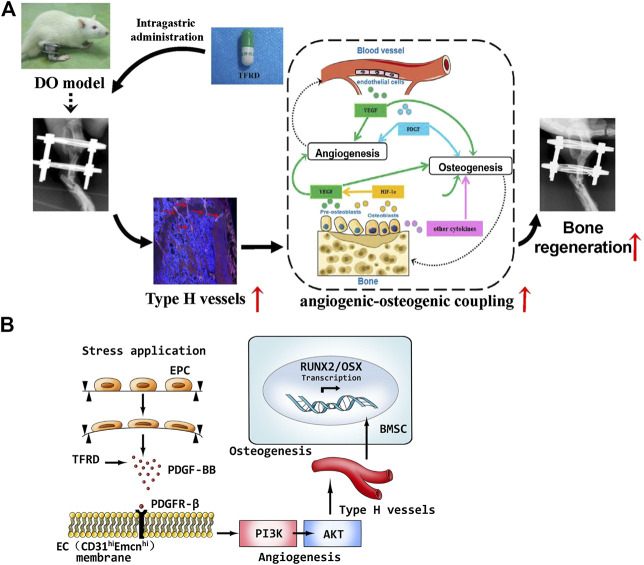
**(A)** Schematic representation of *in vivo* experiments and **(B)** molecular mechanism of TFRD-regulated angiogenic-osteogenic coupling during distraction osteogenesis.

Finally, schematic representation of *in vivo* experiments and underlying molecular mechanism of TFRD-regulated angiogenic-osteogenic coupling during DO were presented in Figure 9. Taken together, administration of TFRD enhances angiogenic -osteogenic coupling *in vivo* during DO by promoting type H vessel formation through PDGF-BB/PDGFR-β signaling axis.

## 4. Discussion

Despite extensive research on the mechanism of DO, little advancement has been made in translating the findings into the clinical application. Nowadays, lack of pharmacotherapies to accelerate bone regeneration in the distraction gap and to permit earlier removal of the fixator remains a major problem that restricts the wide application of DO in clinical practice. Angiogenic-osteogenic coupling plays a significant role during the process of DO, and type H vessels that have been proved to be associated with osteogenesis can promote the coupling (Bernard, 2018). Thus, promoting type H vessel formation and enhancing angiogenic-osteogenic coupling might be an ideal strategy for facilitating DO-induced bone regeneration.

In this study, we first confirmed the presence of type H vessels in the DO model of rats and found more type H vessels in the DO model than in the FR model. This is the first time to report that type H vessels exist in the DO model and regular tensile stress could stimulate the formation of type H vessels. Meanwhile, the *in vivo* results including radiographic, histological, immunohistochemical and immunofluorescent analyses demonstrated that groups with higher abundance of type H vessels showed better angiogenic and osteogenic outcomes, which may be associated with distinct molecular properties of type H vessels that mediate the bone vasculature growth, provide angiocrine signals, generate distinct metabolic and molecular microenvironments and maintain perivascular osteoprogenitors (Kusumbe et al., 2014; Ramasamy et al., 2014; Bernard, 2018). Consistent with the previous study (Gosain et al., 2004), *in vivo* experiments showed more formed bone and vessels in DO procedure than in simple fracture healing process, which indicated tensile stress was beneficial to angiogenesis and osteogenesis. More importantly, TFRD was found to exert positive effects on type H vessel formation and increased the abundance of type H vessels in DO and FR models, which accords with the *in vitro* finding that exposure of EPCs to TFRD could enhance angiogenesis under both stress and non-stress conditions. Interestingly, the immunofluorescent results indicated that the formation of type H vessels in DO and FR groups decreased over time and the abundance of type H vessels displayed the temporal specificity during the DO process, which was similar to the report by Jimeng Wang (Wang et al., 2017), showing a time-dependent decline in the formation of type H vessels. However, in our study, the decrease of type H vessel abundance was alleviated by administration of TFRD.

It has been proved that both HIF-1α/VEGF (Schipani et al., 2009) and PDGF-BB/PDGFR-β (Heldin et al., 2018) signaling play important roles in physiological and pathological angiogenesis, but which signaling regulates the production of type H vessels in the DO model remained elusive. In this study, we noticed that the expression level of serum PDGF-BB showed a similar trend to the temporal change characteristics of type H vessel formation in DO and FR groups.

In addition, EPCs that would develop into type H vessels could secrete PDGF-BB, and the content of PDGF-BB secreted from EPCs could be significantly increased by TFRD in a dose-dependent manner. Therefore, we hypothesized that PDGF-BB involved in the formation of type H vessels and TFRD might promote the formation of type H vessels through PDGF-BB-related signaling pathway during the process of DO. In our tube formation assays, the results exhibited that 100 μg/ml TFRD obviously increased the angiogenesis of EPCs under both stress and non-stress conditions. However, of note, function blocking anti-PDGF-BB antibody significantly inhibited the angiogenesis of EPCs under stress conditions alone, which indicated that mechanical stress stimulation is essential for PDGF-BB-mediated angiogenesis of EPCs, while angiogenesis in simple fracture healing process without mechanical stress stimulation may not be induced by PDGF-BB. To the best of our knowledge, this study is the first to show that TFRD can promote angiogenesis of EPCs that would develop into type H vessels through increasing PDGF-BB under stress conditions. Although previous studies (Anuja et al., 2010; Chen et al., 2011) reported the correlation between PDGF-BB and type H vessels, PDGF-BB was reported to be produced by preosteoclasts in osteoporosis models, which differs from PDGF-BB production from EPCs in the DO model in this study. Furthermore, it is well known that huge differences exist in the molecular or cellular events associated with bone formation between osteoporosis models and DO models. As reported by Lee et al. (Lee et al., 2008; Lee et al., 2010), mechanical stimulation could mobilize EPCs from the bone marrow into the peripheral blood, home them to the distraction gaps and contribute to angiogenesis and bone regeneration, which demonstrated that EPCs were closely associated with the DO process (Lee et al., 2008; Lee et al., 2010).

In order to exclude the role of HIF-1α signaling pathway and further investigate the mechanism of TFRD-promoted type H vessel formation during the DO process, YC-1, the HIF-1α inhibitor, was administrated to the stretched EPCs that simulate type H vessel formation during the DO process. Our results clearly showed that in addition to increased levels of *p*-PDGFR-β, HIF-1α and VEGF, TFRD also up-regulated the levels of *p*-AKT and *p*-ERK1/2 in the stretched EPCs. AKT and ERK1/2 are the primary downstream mediators of the well-known PDGF-BB pathway (Heldin et al., 2018), and previous studies have demonstrated that the PI3K/AKT and MEK/ERK pathways involved in angiogenesis or osteogenesis (Lee et al., 2006; Zhang et al., 2016). The effects were blocked by function blocking anti-PDGF-BB antibody and the corresponding tube formation was also suppressed. However, the activated AKT and ERK1/2 and the tube formation were not affected by the addition of HIF-1α inhibitor, which suggested that TFRD enhanced angiogenesis of the stretched EPCs via PDGF-BB/PDGFR-β rather than HIF-1α/VEGF axis.

Besides, to confirm the presence of cell to cell communication between vascular cells and osteogenic cells during the process of bone formation, we detected the osteogenic differentiation of BMSCs with the treatment of conditioned media (CM) from the stretched EPCs pretreated with TFRD or DMSO. As reported by the previous studies (Grellier et al., 2009; Stiehler et al., 2009; Liu et al., 2014), mechanical stress stimulation influenced the osteogenic differentiation of MSCs through inducing the expression of osteogenic genes and the activity of ALP, OCN and Runx2, which was also confirmed by our study. Our results demonstrated that the stress conditions contributed to more ALP positive nodes and calcium nodes and higher expressions of RUNX2 and OSX at the mRNA and protein levels than the non-stress conditions. Moreover, the CM(TFRD) treatment could significantly promote the osteogenic differentiation of BMSCs and increased the levels of RUNX2 and OSX under both stress and non-stress conditions. But interestingly, blocking PDGF-BB was found to inhibit the osteogenic differentiation of BMSCs only under stress conditions, which indicates that mechanical stimulation is also essential for PDGF-BB-mediated osteogenic differentiation of BMSCs. However, under non-stress conditions, what factor from EPCs mediated the osteogenic differentiation of BMSCs remains unclear, and further study is required.

Taken together, our study confirms the presence of type H vessels in the rat DO model for the first time. Furthermore, TFRD may augment type H vessel formation and subsequently enhance angiogenic-osteogenic coupling during the DO process via PDGF-BB/PDGFR-β instead of HIF-1α/VEGF axis, which suggests that type H vessels may be further developed into a novel therapeutic target for DO and TFRD may represent a promising drug for facilitating bone regeneration in DO by increasing type H vessels through activating PDGF-BB/PDGFR-β signaling axis.

## Data Availability Statement

All datasets generated for this study are included in the article/Supplementary Material.

## Ethics Statement

The animal study was reviewed and approved by Animal Ethic Committee of the First Affiliated Hospital of Guangzhou University of Traditional Chinese Medicine.

## Author Contributions

The concept of the research was provided by ZS and ZJ. ZS, YZ, and ZL contributed to the design of study protocol. Animal surgery was carried out by ZS, YZ, HL, MH, HC, TJ, and ZL. Cell experiments were performed by ZS, YZ, TJ, ZL, and JF. Phenotypic identification of rat BMSCs and EPCs, and differentiation capacities of BMSCs were performed by ZC. Data acquisition was done by ZS, YZ, and ZC. Statistical analysis was done by HL and ZJ. Manuscript was drafted by ZS. All authors read and approved the final manuscript.

## Funding

This study is supported by the National Natural Science Foundations of China (No.81603640 No.81774337 and No.81974575), the Doctoral Fund Project of Kunming Municipal Hospital of Traditional Chinese Medicine, the Joint General Project of Yunnan College of Traditional Chinese Medicine (2017FF117(-048)) and the Supporting Project of Yunnan Provincial Orthopedics Research Center of Integrated Traditional Chinese and Western Medicine (2016NS301).

## Conflict of Interest

The authors declare that the research was conducted in the absence of any commercial or financial relationships that could be construed as a potential conflict of interest.
