# Supplementary material for: Total Flavonoids of Rhizoma Drynariae Enhances Angiogenic-Osteogenic Coupling During Distraction Osteogenesis by Promoting Type H Vessel Formation Through PDGF-BB/PDGFR-β Instead of HIF-1α/ VEGF Axis
Source: Front Pharmacol. 2020 Nov 27;11:503524. doi: 10.3389/fphar.2020.503524 (PMC7729076; doi:10.3389/fphar.2020.503524)
Supplement: Supplementary file 1 [file datasheet1.doc]

Supplementary Material

#
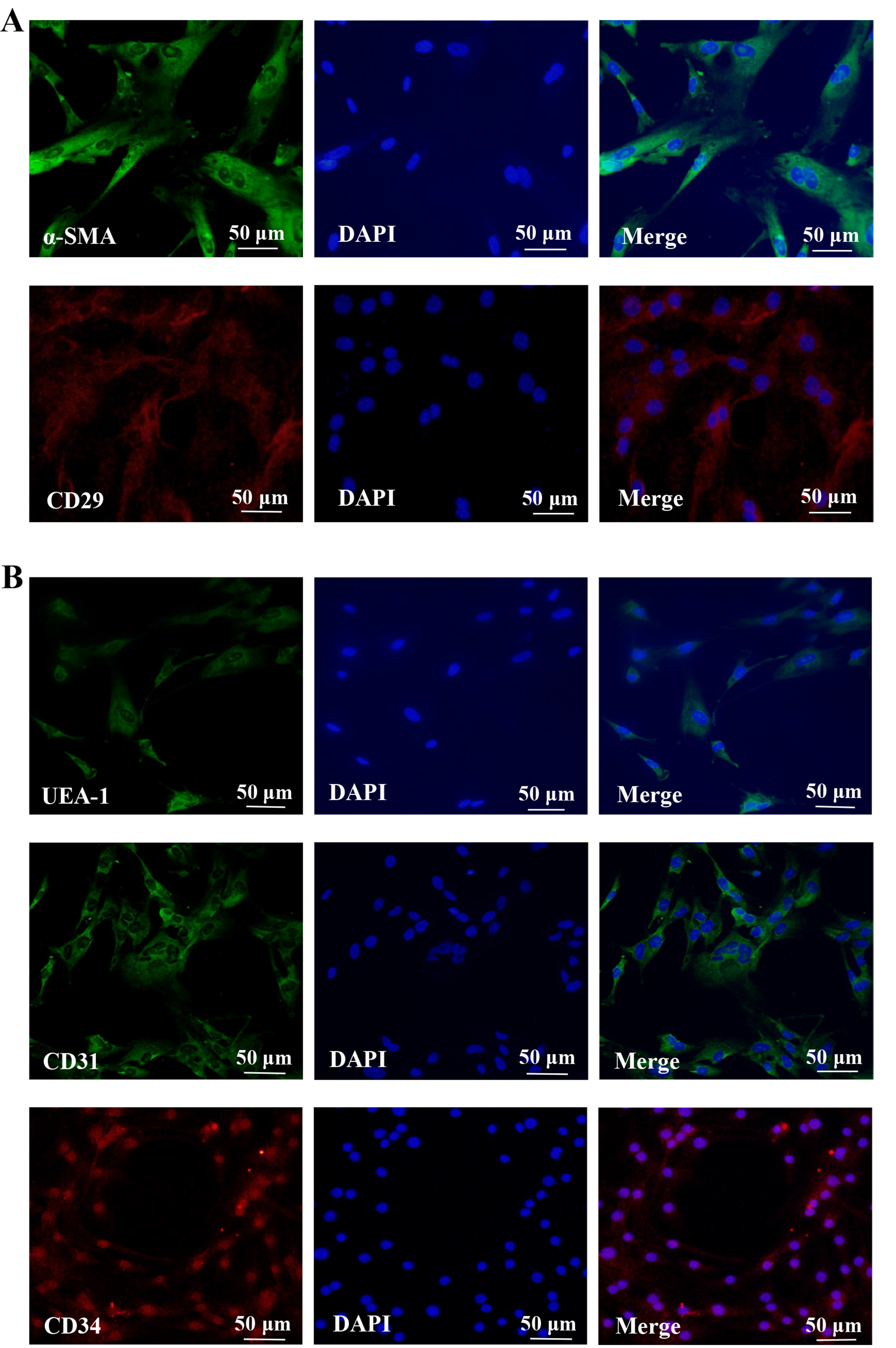


**Figure S1.** Identification of rat BMSCs and EPCs. (A) BMSCs were identified by immunofluorescence staining with α-SMA and CD29, specific markers for BMSCs. Scale bar = 50 μm. (B) EPCs were identified by immunofluorescence staining with UEA-1, CD31 and CD34. Scale bar = 50 μm.


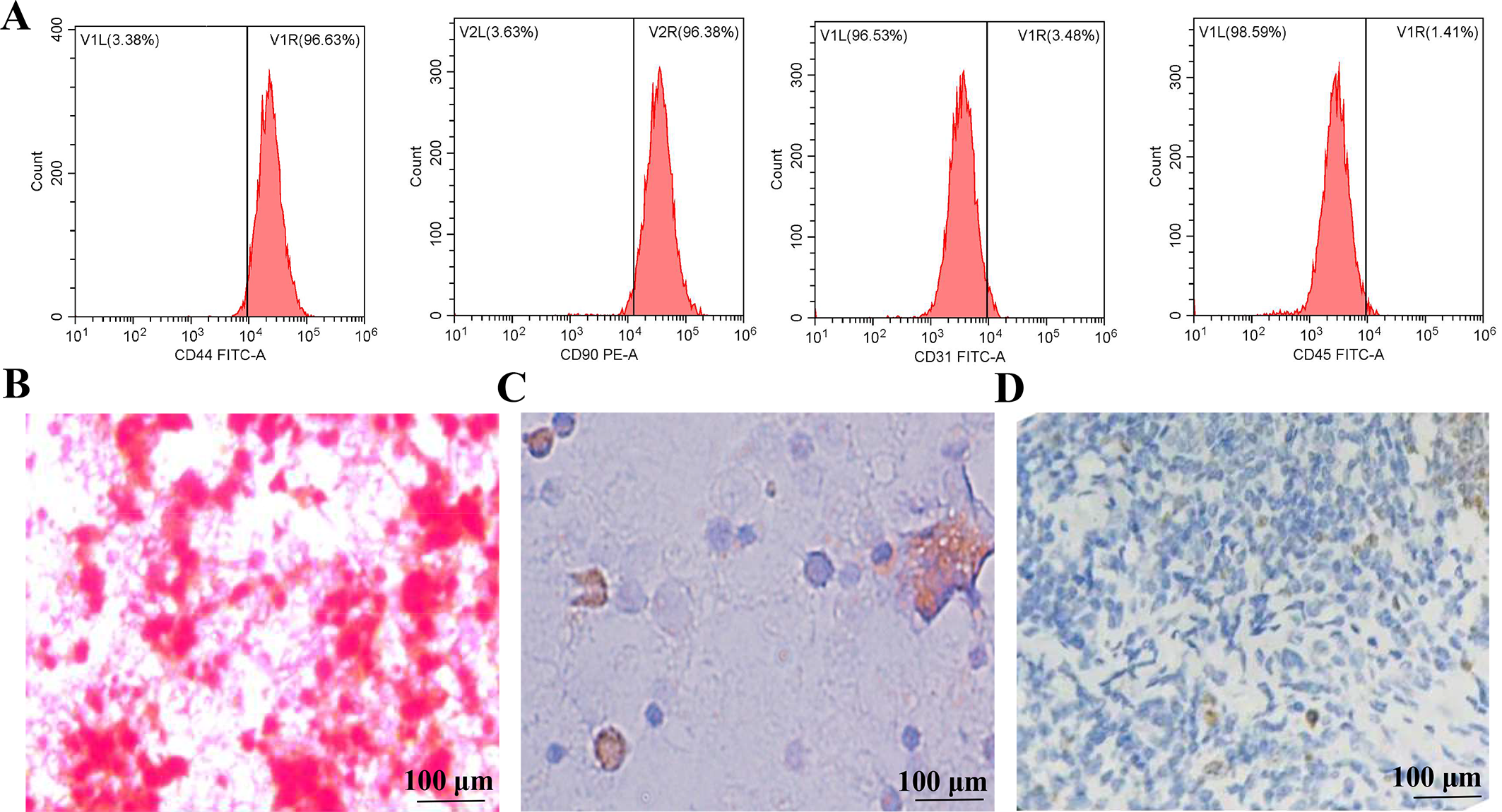


**Figure S2.** Phenotypic identification and differentiation capacities of BMSCs. (A) Flow cytometry analysis results showed that the cells were positive to CD44 (96.63%) and CD90 (96.38%), but negative to CD31 (3.48%) and CD45 (1.41%). (B) Osteogenic differentiation was confirmed by Alizarin Red S staining 14 days after osteogenic induction. Scale bar=100 μm. (C) Adipogenic differentiation was confirmed by Oil Red O staining 14 days after adipogenic induction. Scale bar=100 μm. (D) Chondrogenic differentiation was confirmed by toluidine blue staining 14 days after chondrogenic induction. Scale bar=100 μm.

Table S1. Sequences of primers.

| **Gene** | **Primer sequences (5’-3’)** | |
| --- | --- | --- |
| RUNX2 | Forward | GCTTGATGACTCTAAACCTA |
|  | Reverse | AAAAAGGGCCCAGTTCTGAA |
| Osteorix | Forward | GAAGAAGCTCACTATGGCTC |
|  | Reverse | GAAAAGCCAGTTGCAGACGA |
| β-actin | Forward | ATATCGCTGCGCTGGTCGTC |
|  | Reverse | AGGATGGCGTGAGGGAGAGC |

Table S2. Raw data for the Micro-CT analysis in Figure 1E.

| **Group** | **N** | **BV/TV(%)** |
| --- | --- | --- |
| DO(TFRD) | 3 | 50.23±0.49 |
| DO(CON) | 3 | 37.90±0.36 |
| FR(TFRD) | 3 | 35.34±1.07 |
| FR(CON) | 3 | 25.57±0.95 |

*DO, distraction osteogenesis; FR, bone fracture; TFRD, total flavonoids of Rhizoma Drynariae; CON, control; BV/TV, bone tissue volume/total tissue volume; Data are expressed as mean ± SEM.*

Table S3. Raw data for the angiography and immunohistochemistry analyses in Figures 2B and 2D.

| **Group** | **N** | **Vessel area(mm^3^)** | **CD31-positive vessel number** |
| --- | --- | --- | --- |
| DO(TFRD) | 3 | 0.181±0.006 | 23.20±0.82 |
| DO(CON) | 3 | 0.125±0.008 | 15.50±0.58 |
| FR(TFRD) | 3 | 0.120±0.004 | 15.25±1.73 |
| FR(CON) | 3 | 0.052±0.007 | 8.75±1.50 |

*DO, distraction osteogenesis; FR, bone fracture; TFRD, total flavonoids of Rhizoma Drynariae; CON, control; Data are expressed as mean ± SEM.*

Table S4. Raw data for the histological analysis in Figures 3B and 3D.

| **Group** | **N** | **Newly-formed bone area (%)** | **Newly-formed vessel number** |
| --- | --- | --- | --- |
| DO(TFRD) | 3 | 59.75±0.96 | 26.75±0.96 |
| DO(CON) | 3 | 47.50±0.58 | 18.5±0.58 |
| FR(TFRD) | 3 | 48.25±2.22 | 18.75±0.96 |
| FR(CON) | 3 | 28.00±1.41 | 9.75±1.26 |

*DO, distraction osteogenesis; FR, bone fracture; TFRD, total flavonoids of Rhizoma Drynariae; CON, control; Data are expressed as mean ± SEM.*

Table S5. Raw data for the type H vessel abundance of DO and FR models at day 17 and 45 in Figures 4B and 4D.

| **Group** | **N** | **Type H vessel abundance (%)** | |
| --- | --- | --- | --- |
|  |  | **Day 17** | **Day 45** |
| DO(TFRD) | 3 | 4.61±0.03 | 1.54±0.06 |
| DO(CON) | 3 | 2.38±0.02 | 0.97±0.10 |
| FR(TFRD) | 3 | 2.30±0.02 | 0.09±0.01 |
| FR(CON) | 3 | 0.63±0.04 | 0.04±0.01 |

*DO, distraction osteogenesis; FR, bone fracture; TFRD, total flavonoids of Rhizoma Drynariae; CON, control; Data are expressed as mean ± SEM.*

Table S6. Raw data for the levels of angiogenesis-related factors in serum in Figures 6A-C.

| **Group** | **N** | **HIF-1α (pg ml^-1^)** | | **VEGF (pg ml^-1^)** | | **PDGF-BB (pg ml^-1^)** | |
| --- | --- | --- | --- | --- | --- | --- | --- |
|  |  | **Day 17** | **Day 45** | **Day 17** | **Day 45** | **Day 17** | **Day 45** |
| DO(TFRD) | 3 | 99.18±4.53 | 3.21±0.43 | 236.19±13.01 | 104.36±7.76 | 763.33±18.93 | 268.17±20.63 |
| DO(CON) | 3 | 66.67±3.06 | 5.96±0.96 | 184.80±7.01 | 78.93±2.72 | 381.67±20.21 | 122.33±15.63 |
| FR(TFRD) | 3 | 80.33±6.45 | 51.83±7.78 | 175.69±10.67 | 146.60±8.64 | 377.33±16.17 | 35.00±7.56 |
| FR(CON) | 3 | 18.48±1.03 | 35.38±3.53 | 132.95±6.57 | 103.85±4.52 | 185.17±16.13 | 30.67±8.51 |

*DO, distraction osteogenesis; FR, bone fracture; TFRD, total flavonoids of Rhizoma Drynariae; CON, control; Data are expressed as mean ± SEM.*

Table S7. Raw data for the tube formation parameters of EPCs in Figure 7A.

| **Group** | **Total tube length (px)** | | **Total branching points** | | **Total loops** | |
| --- | --- | --- | --- | --- | --- | --- |
|  | **unstrained** | **strained** | **unstrained** | **strained** | **unstrained** | **strained** |
| CON | 6723.33±146.40 | 10053.33±211.98 | 17.33±1.16 | 33.33±1.53 | 7.33±0.58 | 16.33±1.00 |
| anti-PDGF-BB | 6130.00±147.31 | 1940.67±208.81 | 16.33±0.58 | 5.33±0.58 | 6.33±0.58 | 1.33±0.58 |
| TFRD | 8363.33±127.41 | 13466.67±416.33 | 23.33±0.58 | 46.33±1.3 | 10.33±1.16 | 21.67±1.53 |
| TFRD+anti-PDGF-BB | 7990.00±164.62 | 3706.67±190.09 | 22.67±1.16 | 5.33±0.58 | 9.67±0.58 | 1.67±0.58 |

*TFRD, total flavonoids of Rhizoma Drynariae; CON, control; anti-PDGF-BB, function blocking anti-PDGF-BB antibody; Data are expressed as mean ± SEM.*
